# IGFBP-4: A promising biomarker for lung cancer

**DOI:** 10.5937/jomb0-25629

**Published:** 2021-06-05

**Authors:** Savas Irem Nur, Akin Ozturk, Murat Kavas, Ismet Bulut, Sumeyye Alparslan, Eroglu Selma Aydogan, Baytemir Cansel Atinkaya, Murat Kolay, Abdurrahman Coskun

**Affiliations:** 1 Acibadem Mehmet Ali Aydınlar University, School of Medicine, Department of Biochemistry, Istanbul, Turkey; 2 University of Health Sciences, Istanbul Sureyyapasa Chest Diseases and Thoracic Surgery Training and Research Hospital, Department of Medical Oncology, Istanbul, Turkey; 3 University of Health Sciences, Istanbul Sureyyapasa Chest Diseases and Thoracic Surgery Training and Research Hospital, Department of Chest Disease, Istanbul, Turkey; 4 University of Health Sciences, Istanbul Sureyyapasa Chest Diseases and Thoracic Surgery Training and Research Hospital, Department of Allergy and Immunology, Istanbul, Turkey; 5 University of Health Sciences, Istanbul Sureyyapasa Chest Diseases and Thoracic Surgery Training and Research Hospital, Department of Thoracic Surgery, Istanbul, Turkey; 6 Acibadem Labmed Clinical Laboratories, Department of Biochemistry, Istanbul, Turkey

**Keywords:** lung cancer, biomarkers, IGFBP-4, PAPP-A, IGF-1, karcinom pluća, biomarkeri, IGFBP-4, PAPP-A, IGF-1

## Abstract

**Background:** Insulin-like growth factor binding protein-4 (IGFBP-4), a member of the insulin-like growth factor (IGF) family, transports, and regulates the activity of IGFs. The pregnancy-associated plasma protein-A (PAPP-A) has proteolytic activity towards IGFBP-4, and both proteins have been associated with a variety of cancers, including lung cancer. Thus, we aimed to evaluate the use of IGFBP-4 and PAPP-A as potential biomarkers for lung cancer.

**Methods:** Eighty-three volunteers, including 60 patients with lung cancer and 23 healthy individuals, were included in this study. The patients with lung cancer were selected based on their treatment status, histological subgroup, and stage of the disease. Enzyme-linked immunosorbent assays were used to assess the serum levels of IGFBP-4 and PAPPA, whereas the IGF-1 levels were measured using a chemiluminescent immunometric assay.

**Results:** The serum IGFBP-4 levels in all patient groups, regardless of the treatment status and histological differences, were significantly higher than those in the control group (p<0.005). However, the serum PAPP-A levels in the untreated patient group were found to be higher than those in the control group, but this difference was not statistically significant (p=0.086).

**Conclusions: **The serum PAPP-A and IGFBP-4 levels are elevated in lung cancer. However, IGFBP-4 may have better potential than PAPP-A as a lung cancer biomarker.

## Abstract (sr)

**Uvod:** Vezujući protein 4 (IGFBP-4), član porodice insulinu sličnih faktora rasta (IGF), prenosi i reguliše njihovu aktivnost. Plazma protein A u vezi trudnoće (PAPP-A) ima proteolitičku aktivnost prema IGFBP-4, a oba proteina su povezana sa različitim vrstama raka, uključujući karcinom pluća. Stoga smo želeli da procenimo upotrebu IGFBP-4 i PAPP-A kao potencijalnih biomarkera za karcinom pluća.

**Metode:** U istraživanje je uključeno 83 dobrovoljnih učesnika, uključujući 60 pacijenata sa karcinomom pluća i 23 zdrave osobe. Pacijenti sa karcinomom pluća odabrani su na osnovu njihovog statusa lečenja, histološke podgrupe i stadijuma bolesti. Enzimski imunosorbentni testovi korišćeni su za procenu nivoa seruma vezujućeg proteina 4 i plazma proteina A, dok su nivoi IGF-1 mereni korišćenjem hemiluminescentnog imunometrijskog testa.

**Rezultati:** Nivoi seruma IGFBP-4 u svim grupama pacijenata, bez obzira na status lečenja i histološke razlike, bili su značajno viši od onih u kontrolnoj grupi (p<0,005). Međutim, nađeno je da su nivoi seruma PAPP-A u nelečenoj grupi pacijenata viši od onih u kontrolnoj grupi, ali TA razlika nije bila statistički značajna (p=0,086).

**Zaključak: **Serumski nivoi PAPP-A i IGFBP-4 povišeni su kod karcinoma pluća. Međutim, IGFBP-4 može imati bolji potencijal od PAPP-A kao biomarker raka pluća.

## Introduction

Among all the different types of cancer, lung cancer is the most frequently diagnosed one (2.1 million new cases per year) and is also responsible for most cancer-related deaths (1.7 million deaths per year) [1]. Lung cancer progresses quietly and insidiously, and the majority of patients are often only diagnosed at an advanced stage. Considering all stages, the 5-year survival rate for patients with lung cancer is approximately 17% [2]. The overall 5-year survival rate for patients with lung cancer increases when the disease is detected at an early stage, but unfortunately, only 15% of patients are diagnosed at early stages [2]. Lung cancer is diagnosed by physical examination, radiology, and pathology. Although sputum cytology and chest X-rays are common methods for diagnosis, they are often insufficient [3]. Surgical biopsy, transthoracic needle biopsy, and computed tomography (CT) provide better results than the methods mentioned above. Nowadays, CT is seen as the most effective method for early diagnosis. The sensitivity of this method is 88.9% for low-dose CT, whereas it is only 78.3% for chest X-rays. However, exposure to radiation is a significant disadvantage in CT [3] [4].

Therefore, there is a need for non-radiative and non-invasive diagnostic methods that can detect lung cancer early. Cancer biomarkers are expected to exhibit good diagnostic power in the early phases of diseases and to assess a patient's metastasis status and prognosis [4]. Although there are various biomarkers available for other types of cancer, currently only a limited number of molecules such as carcinoembryonic antigen (CEA), CYFRA 21-1, and neuron-specific enolase (NSA) are used in clinical practice for lung cancer. The serum levels of NSA are elevated in the patients with small-cell lung cancer [5], and the levels of CYFRA-21-1 and CEA are increased in the patients with non-small cell lung cancer [6] and adenocarcinoma [7], respectively. However, these molecules are not lung-specific and are synthesised by both healthy and diseased tissues at different rates [4]. Despite the significant differences in the efficiency of these biomarkers in different histologically defined subtypes of lung cancer, none of these biomarkers is specific to a particular subtype of lung cancer. Although they have positive predictive values in clinical practice, the use of these markers alone is not sufficient for diagnosis because of their low specificity and sensitivity [7] [8]. The inadequacy of these biomarkers has, therefore, led to a search for new, more specific, and sensitive biomarkers. Understanding the molecular mechanism underlying lung cancer progression is essential to develop diagnostic methods in order to provide effective diagnosis, monitoring, and treatment.

The insulin-like growth factor (IGF) family consists of IGFs (IGF-1, IGF-2), IGF receptors, IGF binding proteins 1-6 (IGFBP1-6), and proteases of IGF binding proteins. The six main binding proteins bind IGFs with high affinity, and, besides, there are ten related proteins (IGFBP-rP1-10) that bind IGFs with low affinity [9]. IGFBPs bind to free IGFs and limit their activity [10]. This system regulates a number of important processes, including growth, differentiation, and cell migration [10]. IGFBP-4 is a multifunctional protein that has IGF-independent activities [11] in addition to its IGF binding role. It was first discovered in a human osteosarcoma cell line [12] but is also expressed in embryonic tissues, fibroblasts, prostate, and ovarian cells [11]. IGFBP-4 is degraded by a specific protease referred to as pregnancy-associated plasma protein-A (PAPP-A). IGFs, which are released after PAPP-A-mediated proteolysis of IGFBP-4, can then bind to their receptors to regulate the metabolic activities of their target cells and tissues [13]. The levels of both IGFBP-4 [14] and PAPP-A [13] are known to be elevated in various tumourderived cells and play important roles in various cancers through the IGF system. Studies linking PAPP-A and cancer have shown that PAPP-A is highly expressed and enhances the activities of IGFs in various cancer types [15]. In contrast, IGFBP-4 is involved in tumour growth regulation by inhibiting the activities of IGFs [14]. Additionally, IGFBP-4 appears to be epigenetically silenced in some adenocarcinoma cells, resulting in the suppressed IGF inhibition [16].

Long non-coding RNAs (lncRNAs) are implicated in gene regulation either at the transcriptional or posttranscriptional level [17]. In cancer tissues, mutations and/or abnormal expression of lncRNAs can contribute to tumour growth and metastasis [18]. Recently, it has been found that in lung cancer tissues, lncRNA of IGFBP4-1 is overexpressed and reprograms the energy metabolism, promoting cancer proliferation and metastasis [19].

PAPP-A was recently proposed as a potential lung cancer biomarker [15] [20] [21]. Although the elevated serum levels of PAPP-A have been found in patients with lung cancer [10], IGFBP-4 has not been adequately evaluated as a biomarker for lung or other types of cancer. Accumulating evidence indicates that IGFBP-4 and PAPP-A are significantly involved in cancer development, but the evidence is insufficient. Based on this, we aimed to evaluate both IGFBP-4 and PAPP-A as potential lung cancer biomarkers and to investigate the serum levels of these molecules in different histological types of lung cancer.

## Materials and Methods

### Study Population

This study was carried out at the Acıbadem Mehmet Ali Aydınlar University and Istanbul Sureyyapasa Chest Diseases and Thoracic Surgery Training and Research Hospital. Twenty-three healthy individuals and 60 patients with lung cancer were enrolled in the study. The patient population consisted of various patients with different histological subtypes (squamous cell carcinoma (SC), small cell carcinoma (SCC), adenocarcinoma (AC), neuroendocrine carcinoma, and mixed types) and stages (I-IV) of lung cancer. Both newly diagnosed and patients undergoing treatment (chemotherapy and/or radiotherapy) were included in this study. Patients with a specific histological type were staged according to the current 8^th^ TNM staging system [22]. Pregnancy, cardiovascular diseases, renal failure, asthma, and diabetes were used as exclusion criteria. Detailed characteristics of the patient population are presented in Table 1. In addition, the control group included 11 females and 12 males with a mean age of 48 years (ranging from 41-78).

**Table 1:** Patient characteristics

	Treated Patients (TP)	Untreated Patiens (UTP)
N	36	24
Age, m (max-min)	60 (41-78)	62.5 (46-75)
Male/Female	28/8	21/3
Histology of lung cancer, n (%)		
Adenocarcinoma	8 (%22.2)	9 (%37.5)
Squamous cell carcinoma	15 (%41.7)	6 (%25)
Small cell carcinoma	10 (%27.8)	8 (%33.3)
Others	3 (%8.3)	1 (%4.2)
Stages, n (%)		
Stage IV	20 (%55.6)	14 (%58.3)
Stage III	11 (%30.6)	6 (%25)
Stage II	2 (%5.6)	2 (%8.3)
Stage I	3 (%8.3)	2 (%8.3)
Smokers, n (%)	24 (%67.7)	16 (%67.7)

The study was approved by the Acıbadem Mehmet Ali Aydınlar University Ethics Committee (ATADEK) (Approval no. 2016-16/15). Written informed consent was obtained from each individual included in the study.

### Biochemical Analysis

Blood samples (5 mL) were collected from all participants at around 9:00 a.m. and centrifuged at 1500 x *g* for 10 minutes. The centrifuged serum samples were transferred into two separate Eppendorf tubes and stored at -80°C until analysis.

Chemiluminescent immunometric analysis (Immulite 2000, Siemens Diagnostics Llanberis, Gwynedd, UK) was used to measure the IGF-1 levels. The limit of detection (LOD) of the assay was 13.3 ng/mL, and the coefficient of variation (CV) was 3.77% at 60.3 ng/mL. The IGFBP-4 levels were measured using sandwich enzyme-linked immunoassays (AnshLabs, Webster, TX, USA). The LOD of the assay was 4.74 ng/mL, and the CV was 1.68% at 122.33 ng/mL. An ultrasensitive sandwich enzyme-linked immunoassay (DRG Instruments, Marburg, Germany) was used to measure the PAPP-A levels. The LOD of the assay was 0.023 ng/mL, and the CV was 6.86% at 11.96 ng/mL.

### Statistical Analysis

The Shapiro-Wilk test was used to determine the normality of the data. Since some of the groups did not fit a normal distribution, these data were displayed as the median and interquartile range (IQR). Statistical difference analyses were performed using ANOVA and Kruskal Wallis tests. A parametric post hoc Tukey test and a non-parametric Mann-Whitney U test were used to identify different groups accordingly. Correlation analyses were performed with either the Pearson or Spearman correlation analysis, as appropriate, for all the parameters examined in the patient and control groups. P values of <0.05 indicated statistical significance.

## Results

The serum levels of IGF-1, IGFBP-4, and PAPP-A in treated (TP) and untreated patients (UTP) with lung cancer and the control group are shown in Table 2, and the levels in the different histological subgroups are shown in Table 3.

**Table 2:** Serum levels of IGF-1, IGFBP-4, and PAPP-A in treated and untreated patients with lung cancer and in the normal healthy control group Datas given as median and interquartile range, M, (Q1–Q3); TP; Treated patients, UTP; Untreated patients, *: different from control group, p<0.005

	Control (n=23)	TP (n=36)	UTP (n=24)
IGF-1 (ng/mL)	125, (101-140)	141, (117-158.5)	125, (90.3-151.5)
IGFBP-4 (ng/mL)	104.6, (81.8-128.1)	149.4*, (114.4-186.9)	161.6*, (107.8-206.2)
PAPP-A (ng/mL)	2.89, (1.84-4.39)	2.7, (1.36-4.98)	4.04, (2.31-5.84)

**Table 3:** Serum levels of IGF-1, IGFBP-4, and PAPP-A in the normal healthy control group and in patients with different histological types of lung cancer Datas given as median and interquartile range, M, (Q1-Q3); SCC: Small cell carcinoma (SCC); SC: squamous cell carcinoma (SC), and adenocarcinoma (AC), *:different from control group, p<0.005

	Control (n=23)	AC (n=17)	SC (n=21)	SCC (n=18)
IGF-1 (ng/mL)	125, (101-140)	130, (97.5-170.5)	143, (123-175)	119.5, (88.3-144.2)
IGFBP-4 (ng/mL)	104.6, (81.8-128.1)	131.9*, (106.2-231.6)	163.6*, (124-195)	148.5*, (118.7-187)
PAPP-A (ng/mL)	2.89, (1.84-4.39)	3.32, (2.50-5.39)	3.28, (1.56,6.76)	2.57, (1.83,4.86)

### IGFBP-4 levels

**Figure 1:**
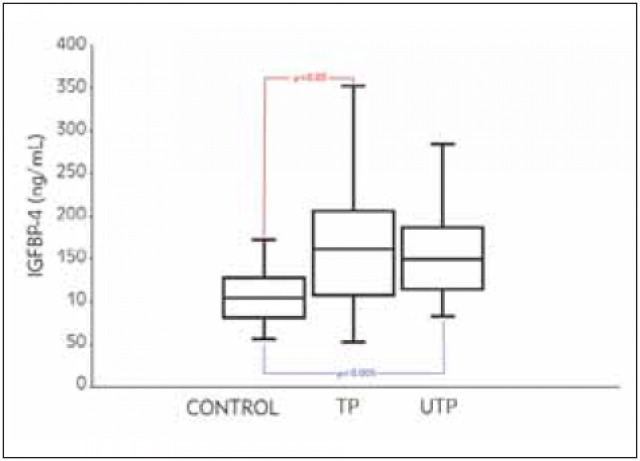
Serum IGFBP-4 levels in the normal healthy control group and the treated (TP) and untreated (UTP) patient groups

The serum IGFBP-4 levels in all patients with lung cancer, regardless of treatment status and histological differences, were significantly higher than those in the control group (Table 2 and Figure 1) (p<0.005). The IGFBP-4 levels in the sera of patients in the SC group were found to be higher than those in both the AC and SSC groups but this difference was not statistically significant (p>0.05) (Table 3). The serum levels of IGFBP-4 were not significantly different among the three histological subgroups (AC, SC, and SCC; p>0.05).

Additionally, for each of the histological subgroups, we also analysed the differences in the IGFBP-4 levels among the patients with different stages of lung cancer, as determined by the TNM staging system. We found no statistically significant differences in the IGFBP-4 levels between the different stages (p>0.05).

### PAPP-A levels

An ultrasensitive PAPP-A ELISA kit was used to measure the PAPP-A levels, and yet 21 (3 AC, 4 SC, 6 SCC, 3 others, and 5 in the control group) out of a total of 83 values were below the LOD.

Although the serum PAPP-A levels in the untreated lung cancer group (4.04 ng/mL) were higher than those in the group of healthy individuals (2.89 ng/mL), the difference was not statistically significant (Table 2) (p=0.086). There were no significant differences between the treated and untreated patients or among the histological subtype groups (Table 2 and Table 3) (p>0.05). In addition, there was no significant difference in the serum PAPP-A levels between the patients with stage IV lung cancer and those with lower stage lung cancer.

### IGF-1 Levels

The serum IGF-1 concentrations did not differ significantly between the different patient groups (TP, UTP, AC, SC, and SCC), and no patient group had the IGF-1 levels that were significantly different from those in the control group (Table 2 and Table 3). Furthermore, there were no significant differences among patients with different stages of lung cancer.

### Correlation Analysis

**Figure 2:**
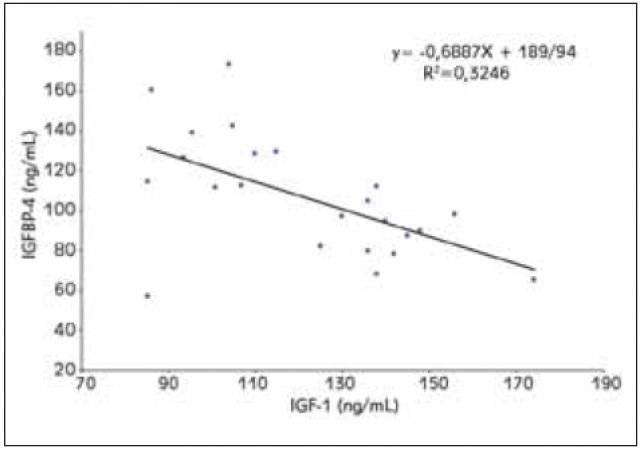
Correlation between IGFBP-4 and IGF-1 levels in the control group

The relationships between the levels of the three different serum proteins (IGF-1, IGFBP-4, and PAPP-A) was examined in all patient and control groups. The serum IGF-1 levels were negatively correlated with those of IGFBP-4 in the control group (Figure 2) (r=-0.57, p<0.05). There was no significant correlation between the levels of any of the three serum proteins in any of the patient groups.

## Discussion

In this study, the IGF-1, IGFBP-4, and PAPP-A levels were analysed in samples from patients with different lung cancer types and stages to evaluate their potential use as lung cancer biomarkers. Our study yielded three main results. Firstly, although not statistically significant, in untreated patients with lung cancer, the serum PAPP-A levels were higher than those in the control group. Secondly, the IGFBP-4 levels were significantly elevated in untreated lung cancer patients compared to those in the control group, suggesting that IGFBP-4 could be a promising biomarker. Thirdly, there was no significant difference in the IGF-1 levels between healthy subjects and subjects with lung cancer.

IGF-1, a physiological regulator of growth in the human body, is involved in tumour metabolism by regulating cell proliferation, clonal growth, and migration [23]. To date, IGF-1 and lung cancer have been found to be associated in various ways [23] [24]. For example, Wang et al. [23] showed that the IGF-1 serum levels were significantly elevated in patients with lung cancer compared to control subjects; however, Lee et al. [24] reported the opposite. In this study, no significant differences in the serum IGF-1 levels were found between healthy control subjects and patients with lung cancer; similar results have been observed in recent studies [25] [26].

In our study, the IGF-1 serum levels in the different patient groups were not statistically different from those in the control group (Table 2). Furthermore, there was no significant difference between stage IV lung cancer patients and patients with lower disease stages (i.e., stages I, II, and III). There was also no significant difference in the IGF-1 levels between patients receiving treatment (chemotherapy and/or radiotherapy) and those not receiving treatment. Izycki et al. [27] did also not observe significant differences in the IGF-1 levels in patients with lung cancer before and after chemotherapy. Previous studies on various cancers, including lung cancer, have emphasised the local synthesis and regulation of IGF-1 in cancer tissues [28]. However, in our study, we measured the IGF-1 levels in sera and not in lung tissues, and therefore measuring the IGF-1 levels in tissues is needed to explore the IGF-1 levels in cancerous and non-cancerous tissues. It should be noted that IGF-1 is one member of the IGF-axis and is regulated by the activity of IGFBPs and their proteases. In our study, we measured the serum levels of IGFBP-4.

IGFs bind to one of the six IGFBPs [10]. IGFBP-4 is one of these binding proteins and inhibits the activity of IGFs by binding to them with high affinity [29]. Although this binding is supposed to inhibit IGF activities, it is also necessary for the activities of IGFs. IGFBP-4 is proteolytically cleaved by PAPP-A, which releases bound IGFs, allowing them to bind to their receptors and activate insulin-like growth factor receptor (IGFR) pathways [30]. Consequently, IGFBP-4 is essential for the transport and regulation of IGFs, and therefore deprivation of IGFBP-4 negatively affects IGF-1 activities.

In addition to non-cancerous tissues, IGFBP-4 is known to be expressed in different histological types of lung cancer cells, such as adenocarcinoma [16] and NSCLC [15]. The inhibitory effects of IGFBP-4 on IGF have also been observed in tumour cells [14]. In our study, we identified a negative correlation between the IGFBP-4 and IGF-1 levels in the normal healthy control group. This relationship may be caused by the inhibitory effects of IGFBP-4 on IGF-1 (Figure 2). In the patient groups, there was no significant correlation between the IGF-1 and IGFBP-4 levels. A study of lung adenocarcinoma cells has suggested that epigenetically suppressed IGFBP-4 positively affects tumour growth by reducing IGF inhibition [16].

Xiao et al. [14] recently showed high expression levels of IGFBP-4 in lung cancer cell lines. In the same study, IGFBP-4 knockdown in the lung cancer cell line A549 caused a decrease in the mRNA and protein levels of IGFBP-4.

In our study, the IGFBP-4 levels were measured in the serum of healthy individuals and patients with lung cancer. Our findings showed that the serum IGFBP-4 levels of all patient groups, regardless of the treatment (TP and UTP) and histological type (AC, SC, and SCC), were significantly higher than those in the control group. These elevated levels suggest that IGFBP-4 is highly associated with lung cancer. Interestingly, the levels of IGFBP-4 in the different patient groups werenot statistically different from each other.

In addition to the well-known IGF-related roles of IGFBP-4, it regulates tumour growth through IGFindependent mechanisms [31]. Furthermore, Hermani et al. [32] reported that IGFBP-4 influences the growth of cancer cells in the absence of IGF by regulating oestrogen receptor-a activation. Although IGFBP-4 has been proposed to regulate tumour growth by affecting malignant progression and inhibiting colony formation, the mechanisms involved in these IGF-independent functions have not been fully elucidated [33].

A characteristic feature of cancer cells is the reprogramming of energy metabolism to support cancer development and uncontrolled growth [34]. Yang et al. [19] have suggested that lnc-IGFBP-4 may play key roles in the energy metabolism and development of cancer. In the same study, while IGFBP-4 expression was found to be decreased in tissues of lung cancer, the expression levels of lnc-IGFBP-4 were increased compared to those in the control group, and this increase significantly correlated with the disease stage and metastasis status [19].

Considering all these lung cancer-related studies on IGFBP-4, it may be concluded that IGFBP-4 is involved in the regulation of cancer development in various ways and may also be a potential biomarker. However, the activity of IGFBP-4 is regulated by its protease PAPP-A.

Despite its name, PAPP-A is not specific to pregnancy as it is also synthesised in men and non-pregnant women. PAPP-A has a high affinity for IGFBP-4-IGF complexes and is responsible for the proteolysis of IGFBP-4 and partly for that of IGFBP-2 and-5 [10]. Although PAPP-A is thought to have roles independent of the IGF system [35], its most prominent task is the proteolysis of IGFBP-4. In recent years, its role in cancer biology, including lung cancer, has been highlighted.

Bulut et al. [20] showed, for the first time, elevated serum PAPP-A concentrations in patients with lung cancer, and subsequent studies have begun to shed light on the role of PAPP-A in lung cancer [15] [34]. Subsequently, an increase in the number of mitotic cells and a decrease in apoptosis have been observed in lung cancers that overexpress PAPP-A [15]. Furthermore, it has been shown that tumour growth decreases when PAPP-A expression is reduced [36].

In this study, the serum levels of PAPP-A in untreated lung cancer patients were higher than those in the control group, but this increase was not statistically significant (p=0.086).

In circulation, PAPP-A may be in an enzymatically active or an inactive form [37]. Most of the circulating PAPP-A is inactive because it is complexed with the pro-form of eosinophil major basic protein (proMBP), and in addition, stanniocalcin-1 (STC-1) and stanniocalcin-2 (STC-2) are known to be PAPP-A inhibitors [28].

Espelund et al. [37] measured the serum levels of IGFBP-4, PAPP-A, and STC-2 in lung cancer patients and showed a positive correlation between the IGFBP-4 and STC-2 levels. Therefore, an increase in the STC-2 levels may lead to PAPP-A inhibition, which in turn increases the IGFBP-4 levels [38]. Although we did not measure the serum levels of proMBP, STC-1, and-2 in this study, we suggest that the significantly higher levels of IGFBP-4 may be due to the effects of these PAPP-A inhibitors.

Despite these findings, our study has some limitations. The mean age of the healthy subjects was lower than that of the patients with lung cancer. Because the exclusion criteria were also established for the control group, we were unable to identify any perfectly age-matched individuals that did not have diseases covered by these exclusion criteria. While the circulating IGF-1 levels are quite low before birth, they increase during adolescence and reach their highest values in adulthood. Some studies have suggested that the IGF-1 levels are age-dependent and begin to decrease with advancing age, especially after 60 years of age [39]. The IGF-1 levels, which are expected to be higher in lung cancer patients, may decrease in the patient groups due to age; thus, age may be a confounding factor. This consideration can only be addressed by repeating the study with patients and control individuals of similar ages. Another limitation of the study is the low number of recruited subjects. It was challenging to find patients with lung cancer but without comorbidities, which increase the serum PAPP-A levels, such as diabetes and cardiovascular diseases. Additionally, the serum PAPP-A levels of 21 out of 83 subjects were below the quantitation limit of the ultrasensitive PAPP-A ELISA method and were thus excluded from the study. Consequently, the number of data used in the study was considerably reduced.

## Conclusion

Our study was carried out on a variety of lung cancer patients, including treated and untreated patients with different types of cancer histology and different stages. The levels of IGF-1, IGFBP-4, and PAPP-A, which represent a branch of the IGF family, were examined in these different patient groups as well as in healthy individuals. Late diagnosis is one of the main reasons why lung cancer has such a high mortality rate, and potential biomarkers may provide opportunities for early diagnosis. Therefore, the search for useful biomarkers continues. Although PAPP-A has been suggested in many studies as a potential biomarker for lung cancer, our results highlighted IGFBP-4 instead, the serum levels of which were found to be significantly different between lung cancer patients and healthy control individuals. IGFBP-4 may, therefore, be a potential independent biomarker candidate and has possible IGF-independent activities.

In conclusion, we argue that, compared to PAPP-A, IGFBP-4 may be a promising innovative biomarker for lung cancer. Further studies in a larger population sample are necessary to confirm the role of IGFBP-4 as a potential biomarker in lung cancer and to determine whether it is clinically useful or not.

## Acknowledgements

We would like to thank Editage (www.editage.com) for English language editing.

## Funding sources

This research did not receive any specific grant from funding agencies in the public, commercial, or not-for-profit sectors.

## Conflict of interest statement

The authors stated that they have no conflicts of interest regarding the publication of this article.

## List of abbrevations

AC, adenocarcinoma; SC, squamous cell carcinoma; SCC, small cell carcinoma; TP, treated patients; UTP, untreated patients.
